# Effects of the El Niño-Southern Oscillation and seasonal weather conditions on *Aedes aegypti* infestation in the State of São Paulo (Brazil): A Bayesian spatio-temporal study

**DOI:** 10.1371/journal.pntd.0012397

**Published:** 2024-09-12

**Authors:** Monica Pirani, Camila Lorenz, Thiago Salomão de Azevedo, Gerson Laurindo Barbosa, Marta Blangiardo, Francisco Chiaravalloti-Neto

**Affiliations:** 1 MRC Centre for Environment & Health, Department of Epidemiology and Biostatistics, School of Public Health, Imperial College London, London, United Kingdom; 2 Institute of Advanced Studies, University of São Paulo, São Paulo, Brazil; 3 Health Department of the Municipality of Santa Bárbara d’Oeste, São Paulo, Brazil; 4 Pasteur Institute, Secretary of Health of the State of São Paulo, Brazil; 5 Laboratory of Spatial Analysis in Health, Department of Epidemiology, School of Public Health, University of São Paulo, São Paulo, Brazil; University of Oxford, UNITED KINGDOM OF GREAT BRITAIN AND NORTHERN IRELAND

## Abstract

**Background:**

Seasonal fluctuations in weather are recognized as factors that affect both *Aedes* (*Ae*.) *aegypti* mosquitoes and the diseases they carry, such as dengue fever. The El Niño-Southern Oscillation (ENSO) is widely regarded as one of the most impactful atmospheric phenomena on Earth, characterized by the interplay of shifting ocean temperatures, trade wind intensity, and atmospheric pressure, resulting in extensive alterations in climate conditions. In this study, we investigate the influence of ENSO and local weather conditions on the spatio-temporal variability of *Ae*. *aegypti* infestation index.

**Methods:**

We collected seasonal entomological survey data of immature forms of *Ae*. *aegypti* mosquitoes (Breteau index), as well as data on temperature, rainfall and the Oceanic Niño Index (ONI) for the period 2008–2018 over the 645 municipalities of the subtropical State of São Paulo (Brazil). We grounded our analytical approach on a Bayesian framework and we used a hierarchical spatio-temporal model to study the relationship between ENSO tracked by ONI, seasonal weather fluctuations and the larval index, while adjusting for population density and wealth inequalities.

**Results:**

Our results showed a relevant positive effect for El Niño on the *Ae*. *aegypti* larval index. In particular, we found that the number of positive containers would be expected to increase by 1.30-unit (95% Credible Intervals (CI): 1.23 to 1.37) with El Niño events (i.e., ≥ 1°C, moderate to strong) respect to neutral (and weak) events. We also found that seasonal rainfall exceeding 153.12 mm appears to have a notable impact on vector index, leading potentially to the accumulation of ample water in outdoor discarded receptacles, supporting the aquatic phase of mosquito development. Additionally, seasonal temperature above 23.30°C was found positively associated to the larval index. Although the State of São Paulo as a whole has characteristics favourable to proliferation of the vector, there were specific areas with a greater tendency for mosquito infestation, since the most vulnerable areas are predominantly situated in the central and northern regions of the state, with hot spots of abundance in the south, especially during El Niño events. Our findings also indicate that social disparities present in the municipalities contributes to *Ae*. *aegypti* proliferation.

**Conclusions:**

Considering the anticipated rise in both the frequency and intensity of El Niño events in the forthcoming decades as a consequence of climate change, the urgency to enhance our ability to track and diminish arbovirus outbreaks is crucial.

## Author summary

Dengue fever is a dramatically expanding disease transmitted by mosquitoes. Earlier research has demonstrated the susceptibility of dengue-carrying mosquitoes to weather conditions, with rainfall and temperature significantly impacting dengue transmission. While there is evidence of a connection between weather factors, the El Niño-Southern Oscillation (ENSO) and the risk of dengue, their impact on the behaviour of *Aedes* (*Ae*.) *aegypti* mosquitoes, which is the main disease vectors, remains poorly comprehended. In this study we analyse municipality-specific time series of a larval index of *Ae*. *aegypti* population in the State of São Paulo (southern Brazil) from 2008 to 2018. The main goal was to explore how intra- and inter-annual changes in weather conditions and ENSO periodic fluctuations affected mosquito abundance. We found a strong and significant coherence between the temporal pattern of mosquito abundance and El Niño phases, rainfall and temperature, showing the role that climate plays in driving infestation periodicity. Specifically, we identified a relationship between *Ae*. *aegypti* infestation and the strength of the ENSO phenomenon: periods characterized by El Niño phase (moderate to strong) were associated with a higher abundance of *Ae*. *aegypti* mosquito larvae. Additionally, we found that wealth inequalities increase opportunities for *Ae*. *aegypti* proliferation. Our discoveries carry significant implications for vector control strategies, as they offer valuable insights for categorizing areas based on spatial risk, enabling the prioritization of intensified vector control measures. Given the anticipated rise in the frequency of El Niño events due to climate change, these findings suggest that arbovirus outbreaks could also see an increase, and that El Niño occurrences might serve as a predictive tool for such outbreaks.

## Introduction

Arboviruses such as dengue, yellow fever, chikungunya, and Zika have spread widely in tropics and sub-tropics regions in the last few decades, and their public health impact has dramatically increased, overloading the healthcare system and causing serious socio-economic losses. In particular, dengue fever is currently one of the fastest spreading mosquito-borne viral diseases worldwide [1] and represents the greatest human disease burden of any of such diseases [2].

Each year, an estimated 390 million individuals (95% Credible Intervals (95%CI) 284 to 528 million) contract one of the four serologically distinct serotypes of the dengue virus (DENV-1 to -4) [3]. This neglected tropical disease has experienced a 30-fold surge in global incidence over the past five decades, encompassing its severe manifestation, dengue haemorrhagic fever [4]. Prospective studies indicate a significant rise in dengue cases over the next 30 to 60 years [2]. Despite numerous investigations [5], the escalating risk and outbreaks of dengue have persisted, with transmission dynamics being highly variable due to intricate interactions among virus serotypes, vectors, and hosts [1].

Although with few exceptions, the transmission of arboviruses depends on the presence of the competent mosquito vectors mainly of the species *Aedes* (Ae.) aegypti, which are anthropophilic mosquitoes, targeting humans as their preferred blood source [6,7]. It is known that climate strongly affects the ecology of *Aedes* mosquitoes and the virus, mainly the annual changes in rainfall and temperature [8,9]. These variables influence mosquito larval habitat and development, as well as their reproduction and mortality rates, along with the blood feeding frequency of the *Aedes* female and the extrinsic incubation period of the virus. These factors determine the degree and shape of human exposure to that infection [10].

The control of *Ae*. *aegypti* is still considered a major challenge for public health authorities, especially in developing countries [11]. Routinely performed vector control programs still remain the most effective preventive intervention for arthropod-borne diseases [12], since treatments are expensive, and most of the vaccines are still in clinical development.

The El Niño-Southern Oscillation (ENSO), an ocean-atmosphere phenomenon of the Pacific Ocean with a semi-periodic multiannual cycle [13], has been hypothesized to play a pivotal role in fuelling dengue epidemics in vulnerable regions by exerting a profound impact on the local climate [1,14–18]. ENSO is characterized by two main phases: El Niño, which is the period when water in the Pacific region is warmer then the long-term average, and La Niña, which is characterized by unusually colder than average ocean temperatures in the equatorial region of the Pacific Ocean. Between these two phases there is a third phase, called neutral as it is neither El Niño or La Niña (i.e., no obvious warmer or colder than normal waters). These patterns are associated with changes in the tropical climate, increasing or decreasing the occurrence of extreme dry or wet events [19], and impacting the hydrologic cycle.

Numerous studies in Brazil have examined droughts in the Northeast and North regions, focusing on the impact of the ENSO [20,21] and the patterns of Atlantic basin sea surface temperature anomaly [22,23] during such events. However, in the states of Southeastern Brazil, situated in subtropical latitudes and within the transition zone between tropical and extratropical circulations, the influence of these climate patterns on rainfall remains less defined. Specifically, in São Paulo state, there is a noted tendency for rainfall and temperature to increase during the most severe El Niño periods, while decreasing during La Niña periods of greater intensity [24].

El Niño initiates with an increase in surface seawater temperature in the tropical eastern Pacific Ocean, subsequently spreading westward and inducing warming effects on surface air [25]. Elevated air temperatures can enhance the transmission of dengue by accelerating the replication rate of the dengue virus in *Ae*. *aegypti* [26] and influencing the blood-feeding behaviour of this insect. [27]. Studies performed in different areas including the South Pacific Islands [28], Indonesia [29,30], Thailand [31], Sri Lanka [17], Costa Rica [32] and Brazil [33] shown that El Niño is associated with an increase in the reported number of dengue cases, while a recent study on Solomon Islands estimated a risk reduction in dengue-like-illness due to El Niño and an increased risk due to La Niña [34].

However, a few studies from Latin America have evidenced its regional importance [35] and most studies have focused on the effects of ENSO on the dengue disease [e.g.,^15^], but few relate to infestation by the mosquito vector. The main purpose of this study is to examine the seasonal (three months) and geographical relationship between ENSO and weather variability with the *Aedes* mosquito infestation during the period 2008–2018 in a highly endemic dengue region of southeastern of Brazil. Then, we address whether the local climate variability may account for the observed variation patterns of *Ae*. *aegypti* infestation in this subtropical epidemiological setting.

## Methods

### Study area

This spatio-temporal entomological study was carried out in the State of São Paulo, the most populous state of Brazil, located in the southeastern part of the country and with an area of approximately 248.219 km^2^ divided into 645 municipalities, with an average population density of about 179 inhabitants per km2 [36], which is heavily concentrated on the east portion of its territory. S1 Fig (left panel) of the Supplementary Information displays the map of the State of São Paulo, showing the population density for each municipality at the 2010 Brazilian Demographic Census.

The State is one of the most economically important and developed regions of the country and receives thousands of migrants every day. It is predominantly characterized by the Atlantic forest and Cerrado biomes, and has a dominant tropical climate, with rains in the summer and dry weather in the winter [37].

### Entomological survey data

Following the reintroduction of the *Ae*. *aegypti* species in the 1980s [38], current information on vector infestation was obtained through the State Endemic Control Superintendence (SUCEN). Entomological information from the Rapid Survey of the *Ae*. *aegypti* Infestation Index (LIRAa) was used for the analyses. LIRAa has the advantage of quickly and accurately presenting indices of larval infestations by performing searches for immature forms of the vector in a sample of buildings [39]. In this study, we considered the Breteau index that is computed as the number of positive recipients over the number of properties surveyed, expressed per 100 properties, that is, BI = Positive Containers x 100 / Properties Searched [39,40,41]. Among the different immature *Aedes* indexes used for entomological surveillance, the Breteau index is considered to be the most informative as it establishes a relationship between positive containers and premises [17,42]. The entomological data collection was conducted using a proportional random sample of blocks, accounting for the number of buildings in each block. All buildings within each sampled block, except the ones that were closed or refused the visit of the dengue control agents, were searched for immature forms of mosquitoes. During the visits to the buildings, the agents identified each container with water and collected, when present, mosquito larvae, which were deposited in a glass tube, so that each tube corresponded to a container. Then, these tubes were sent to the Entomological Laboratories for larval identification, and those positive for *Ae*. *aegypti* corresponded to the positive breeding sites, which were considered for the calculation of the Breteau indices [43]. The Brazilian Ministry of Health recommends that Brazilian municipalities with the presence of *Ae*. *aegypti* carry out the LIRAa at least four times a year, based on the National Dengue Control Programme guidelines [39,44]. Nonetheless, this guideline is not always followed and depends on the availability of resources in each of the municipalities. Two of the 645 municipalities of the State of São Paulo (Campos de Jordão, and Rio Grande da Serra) have not reported the *Ae*. *aegypti* presence. Nevertheless, we underline that the lack of collection records from these municipalities should not be strictly interpreted as the absence of the mosquitoes, especially if the larval mosquitoes have been collected from nearby areas.

All LIRAa measurements performed during the study period (2008 to 2018) for each municipality were included in this study. We organized the data in quarterly series per year, corresponding to the cyclic annual seasons during the years 2008–2018; that is: Quarter 1 (Q1) represents January to March, Quarter 2 (Q2) April to June, Quarter 3 (Q3) July to September, and Quarter 4 (Q4) October to December. We chose to aggregate the values of the Breteau index over a three-month period, due to the sparsity of the entomological data, as municipalities exhibited significant gaps in monthly Breteau index data. This is largely attributable to the typical survey frequency in municipalities, which is generally bimonthly or quarterly. If the municipality had measured the Breteau index two or three months in a season and year, we considered their average; if the municipality had measured it only one time, we considered that value; if the municipality had not measured the index, we considered it as a missingvalue. In the SI, we provide the plot of missing information by year (S2 Fig).

### Climatic and socioeconomic data

**Fig 1:**
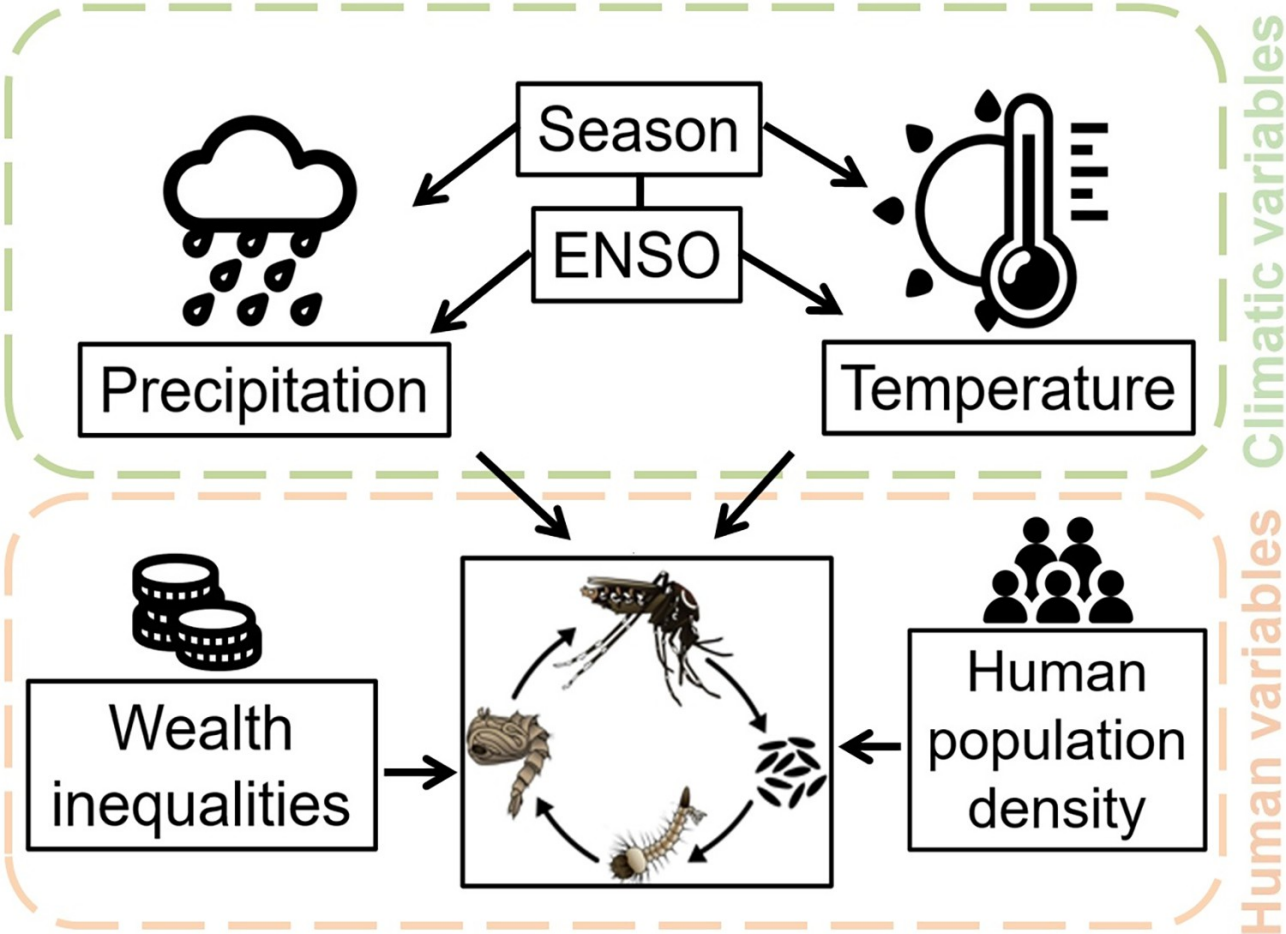
Conceptual framework of the study. The green and pink boxes refer respectively to the climate and human populations variables potentially associated with the mosquito’s life cycle. Source of mosquito images: https://www.cdc.gov/zika/pdfs/spanish/MosquitoLifecycle-sp.pdf CC BY 4.0 license. Source of other icons: https://iconduck.com/ Open-source CC BY 4.0 license.

In our study, we use the National Oceanic and Atmospheric Administration (NOAA)’s Oceanic Niño Index (ONI), which is defined as the 3-month running means of sea surface temperature (SST) anomalies in the east-central tropical Pacific Ocean, as a metric for ENSO. To be classified as a full-fledged El Niño and La Niña phase the ONI must exceed +0.5 (El Niño) or -0.5 (La Niña) for at least five consecutive months [45]. We obtained the data on monthly ONI from the National Institute for Space Research (Instituto Brasileiro de Pesquisas Espaciais) [46]. Being ENSO a global climatic phenomenon that influences weather patterns worldwide, it is important to note that the ONI values, reflecting sea surface temperature anomalies in the central equatorial Pacific, are uniform across all geographical areas at any given time. Therefore, within the context of our study encompassing 645 municipalities, the ONI serves as a time varying factor only. Its values are consistent across all municipalities at any specific point in time, highlighting its role as a temporal climatic driver. Similarly to Anyamba et al. (2019) [15], due to the quarterly aggregation of the data used in this study, for inferential purposes, we adopted a conservative approach and considered |1|ONI value as the threshold instead of the more frequently used |0.5|°C because of the variability in the climate response to ENSO events. Therefore, we classified the ENSO with ONI ≥1°C as El Niño phase, ONI ≤ -1°C as La Niña phase, then -1°C < ONI < 1°C as neutral phase. In addition to ENSO, we considered spatially and temporally-resolved historical weather data, including into the analyses average temperature (°C) and rainfall (mm), which were obtained from the National Institute for Space Research [46] for each seasonal calendar time and municipality. Finally, we adjusted our analyses for municipality-level socioeconomic conditions potentially associated with *Aedes* occurrence [47]. In particular, we retrieved population density (inhabitants/km^2^) from the Brazilian National Institute of Geography and Statistics (IBGE), and the Gini index from IBGE using data from the 2010 Brazilian Demographic Census. Broadly speaking, the Gini index is a measure of income or wealth inequalities and ranges from 0 to 100%, with 0 representing perfect equality and 100 representing perfect inequality. In the State of São Paulo it ranged from 0.33% to 0.67% as shown in S1 Fig (right panel), which displays the different socioeconomic profiles of the municipalities, and reveals a larger vulnerability in the south part of the State. To make the variables comparable, they were standardized by subtracting the mean and dividing by the standard deviation prior being included in the model described in the following section. Fig 1 provides a graphical representation of the conceptual framework of our study, highlighting the variables considered, which are potentially associated to the developmental stages and life cycle of *Ae*. *aegypti*.

### Bayesian spatio-temporal analysis

We propose to analyse the effect of the ENSO, tracked by the NOAA’s ONI, and the local weather conditions on the variability of the *Aedes* larval index using a Bayesian modelling approach, formally incorporating spatio-temporal autocorrelation components in the multilevel structure through the inclusion of random effects, while adjusting for population density and wealth inequalities. Computation is based on the Integrated Nested Laplace Approximation (INLA) [48] and is performed using the R-INLA package [49], which represents a popular framework for performing reliable approximated Bayesian inference (https://www.r-inla.org/).

In the following, we provide a general description of the model, while the details are given in the S1 Text. Note that in our model we did not include a lag structure to describe the relationship between ONI and the weather variables with the larval index due to the organisation of data into seasonal intervals (3 months), performed in order to address the issue of sparsity of the entomological data. Given that the life cycle of the *Ae*. *aegypti* mosquito typically spans 7 to 10 days from larval stage to adulthood [50], our temporal analytical unit (3 months) already encompasses potential late climate influences on mosquito life cycles. Let yst be the *Ae*. *aegypti* larval index collected across space (s = 1, …, S = 645 municipalities) and time (t = 1, …, T = 44 calendar quarter periods). We specified a Negative Binomial data model μst ∼ NegBinom (μst, φ) (controlled by the size parameter φ), with process model:

$$\log(\mu_{st})=\beta_{0}+\sum_{k=2}^{3}\beta_{k}{ONI}_{tk}+f_{1}({Rain}_{st})+f_{2}({Temp}_{st})+\sum_{c=2}^{4}\emptyset_{c}{Season}_{tc}+\gamma_{1}{Density}_{st}+\gamma_{2}{Gini}_{s}+\delta_{st},$$ (1)

Where β0 is the intercept, βk is the coefficient associated with ENSO tracked by ONI (reference category: neutral phase), f1(·) and f2(·) capture nonlinearity in the effect of rainfall [Rain] and temperature [Temp] respectively, ϕc is the coefficient associated with calendar season [Season] (reference category: summer), γ1 and γ2 are the regression coefficients for population density [Density] and the Gini index [Gini] respectively, and δst is a random spatio-temporal effect. We assumed that δst evolves dynamically over time according to an autoregressive process of first order, while at each time point a spatial structure occurs linking the municipalities through an intrinsic conditional autoregressive model [51,52,53,54]. To specify the nonlinear effect of the weather variables (i.e., rainfall and temperature) on the larval index, we binned the values into groups, using equidistant quantiles in their probability space and we modelled these using a random walk priors of first order.

The issue of not-reported larval surveys across municipalities during the period in study was easily handled in R-INLA. In fact, missing response data provide no contribution to the likelihood, while their posterior predictive distributions (PPD) is computed automatically [53,55]. In detail, we set the missing larval surveys to be equal to “NA” and the posterior marginal for the corresponding linear predictor was computed.

To identify unusual high values of the larval index, we computed exceedance probabilities defined as the probabilities of the expected values of the index, estimated for each municipality *s* at quarterly seasonal period *t*, to be greater than a given threshold value *c* [56]. Specifically, for our study we investigated the probability of the posterior estimates of each municipality at time *t* to be equal or larger than 4, as it identifies a condition at risk for *Ae*. *aegypti* infestation based on the National Dengue Control Programme of the Brazilian Ministry of Health [40,57]. The details for the computation of the exceedance probabilities are presented in the S1 Text.

### Model assessment

We used two model selection criteria to identify the best model: (i) the popular Deviance Information Criterion (DIC) [58], which represents a trade-off between model fit and complexity, and (ii) the cross-validated mean logarithmic score (LS) [59], which is a cross-validation measure, where in turn each observation is left out and the PPD is computed based on the remaining observations. Based on these criteria, we contrasted the model presented in Eq 1, with other formulations, whose details are specified in the S1 Text.

In summary, we first built non-spatially and -temporally explicit models including only the measured covariates, without any spatio-temporal random effect. In particular, we modelled the Breteau index against (i) temperature and rainfall (M1), additionally evaluating in turn their potential nonlinear effects (M1a: linear effect of rainfall and nonlinear effect of temperature; M1b: nonlinear effect of rainfall and linear effect of temperature; and M1c: nonlinear effect of rainfall and temperature); (ii) ENSO phases without adjustment for any other covariate (M2). Successively, we evaluated the effect of ENSO phase adjusting for the weather variables (rainfall and temperature) and seasonal period (M3); and then we included all the observed covariates into the model: ENSO phase, rainfall, temperature, seasonal period, population density and Gini index (M4). Finally, we included into the model all the observed covariates and the spatio-temporal structured random effects (M5), as presented in Eq 1. A sensitivity analysis was also performed to check the influence of the choice of priors for the fixed and random effects on the parameter estimates.

## Results

Among the 645 municipalities of the State of São Paulo, 642 had at least one larval survey per season, with 434 measures of the Breteau index in the summers, 289 in the autumns, 412 in the winters, and 456 in springs, on average per year. The yearly proportion of municipalities that did not carry out larval surveys is presented in S2 Fig.

**Fig 2:**
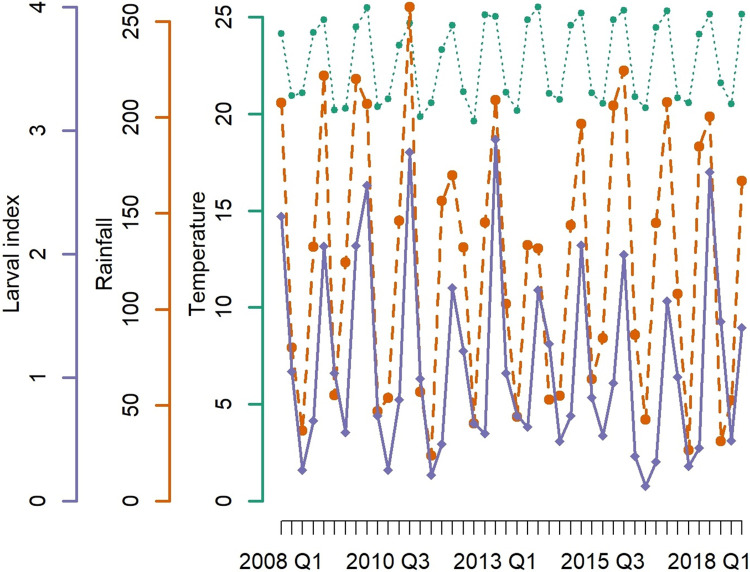
Temporal evolution of the averaged *Ae*. *aegypti* larval index (solid violet line), rainfalls (dashed brown line) and temperature (dotted green line). State of São Paulo, 2008 to 2018.

Fig 2 displays the fluctuation of the larval index, rainfall and temperature across the State of São Paulo for the period in study, showing a pronounced seasonality and inter-annual variability. Summer (Q1) is the season with higher *Ae*. *aegypti* infestation levels, while spring (Q4) represents the period of the year when the infestation level begins to increase. S3 and S4 Figs display the geographical distribution of the collected larval indexes for the spring and summer months respectively of each year, with specification of the ONI index.

**Fig 3:**
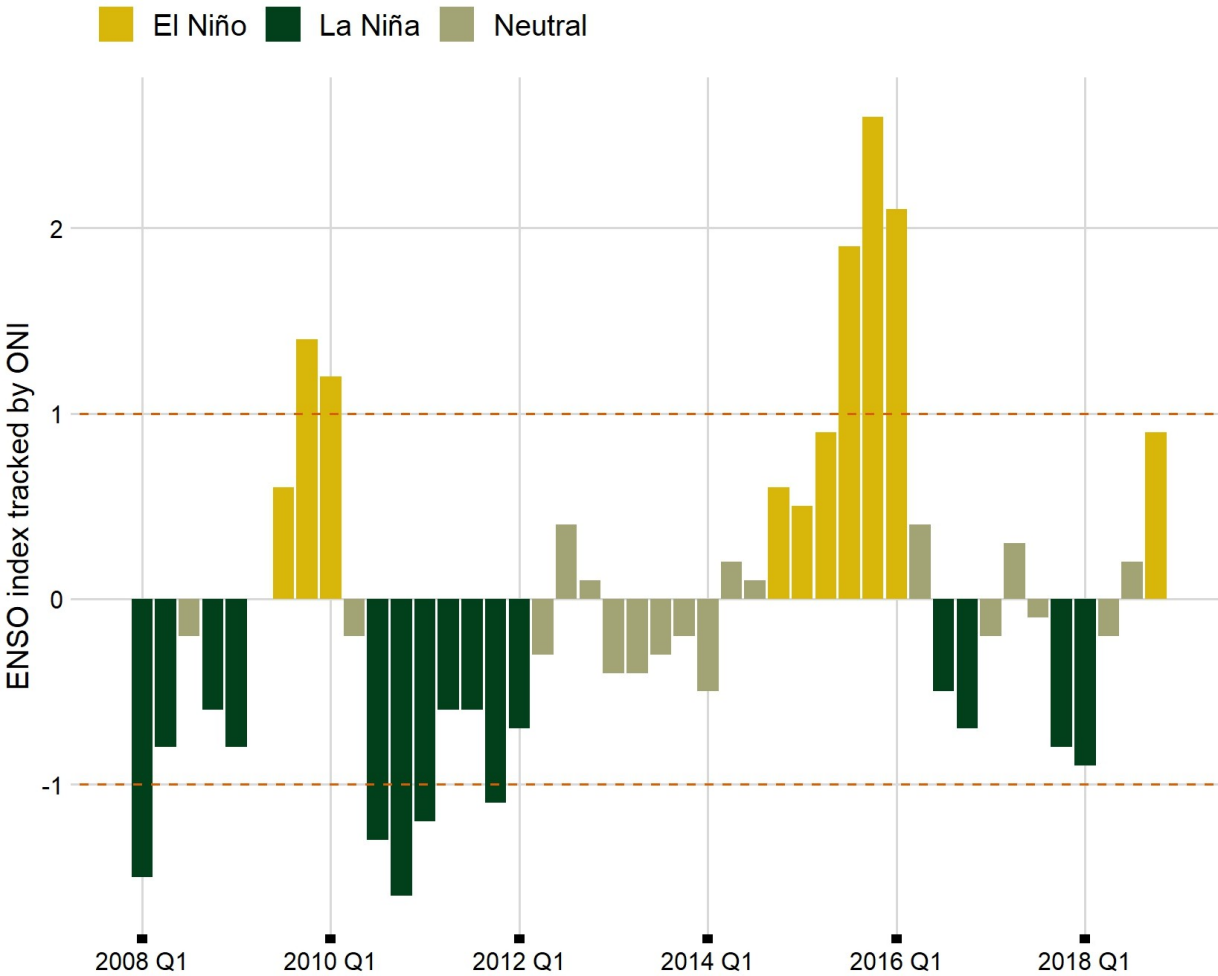
Temporal pattern of El Niño-Southern Oscillation (ENSO) phases recorded by the NOAA’s Oceanic Niño Index (ONI), with colours based on a threshold of ± 0.5°C of ONI, and red dashed lines indicating the threshold used in this study. State of São Paulo, 2008 to 2018.

The average temperature for the period in study was approximately 22°C (range 12.37°C to 35.32°C), but in the hottest months it increased to 25°C, while the seasonal average rainfalls ranged between 0.01 mm to 1104.7 mm with higher incidence in the spring and summer months (for details, see the summary statistics in S1 Table. Fig 3 presents the temporal pattern of the ENSO index based on a threshold of +/-0.5°C for the ONI. During the period in study, there were two important El Niño episodes, which occurred in 2009–2010, 2015–2016, then it started to develop again in 2018, while La Niña events occurred in 2008, 2010–2011 and 2017–2018. As specified in the “Methods” section, for inferential analysis we used a more conservative threshold for ONI of +/- 1°C, similarly to Anyamba and colleagues [15].

Preliminary exploratory analyses, based on Spearman’s rank correlation analysis, showed a statistically positive correlation between the larval index with temperature and rainfalls (respectively, ρ = 0.23 and ρ = 0.28). We also computed the Kruskal-Wallis rank sum test for larval index and the ENSO index, which showed a p-value less than the significance level 0.05, suggesting significant differences between the larval mosquitoes density and the three ENSO phases tracked by ONI. We firstly evaluated the association between the vector index with the climatic variables, then we extended the model to include the socioeconomic covariates and the random space-time effects. The model assessment steps are described in the S1 Text. Here, we present the results from the adjusted model described in Eq 1.

The posterior mean and the 95%CI of the model’s regression coefficients are displayed in Table 1. We found a positive effect for both the moderate to strong phases of La Niña and El Niño on the *Aedes* larval index, although the 95%CI for La Niña included the null value of 1. In particular, our results show that the difference in the number of positive containers would be expected to increase by 1.30 unit (95%CI: 1.23 to 1.37) with El Niño events (i.e., ≤ 1, moderate to strong) respect to neutral (and weak) events. Additionally, we found clear evidence of an association with the seasonal period of the year in the State of São Paulo, and contrasting Autumn, Winter, and Spring against the Summer season, we observed a reduction of the expected values of the *Ae*. *aegypti* larval index.

**Table 1:** Posterior mean and posterior 95% Credible Intervals (on natural scale) for the (fixed) regression parameters associated with the *Ae*. *aegypti* larval index.

Predictor	Mean	2.5%	97.5%
**ENSO tracked by ONI**			
Neutral	1		
La Niña	1.06	1.00	1.13
El Niño	1.30	1.23	1.37
**Season**			
Summer	1		
Autumn	0.81	0.70	0.93
Winter	0.32	0.28	0.37
Spring	0.37	0.35	0.39
**Population density**	1.00	0.88	1.13
**Gini index**	1.42	1.31	1.54

Among the socioeconomic variables considered, we found that an increase of one standard deviation in the Gini index was associated with an increase in the larval index of 1.42 (95%CI: 1.31 to 1.54), while the regression coefficient for population density, although showing a positive association with the larval index, presented 95%CI crossing the null value of 1.

**Fig 4:**
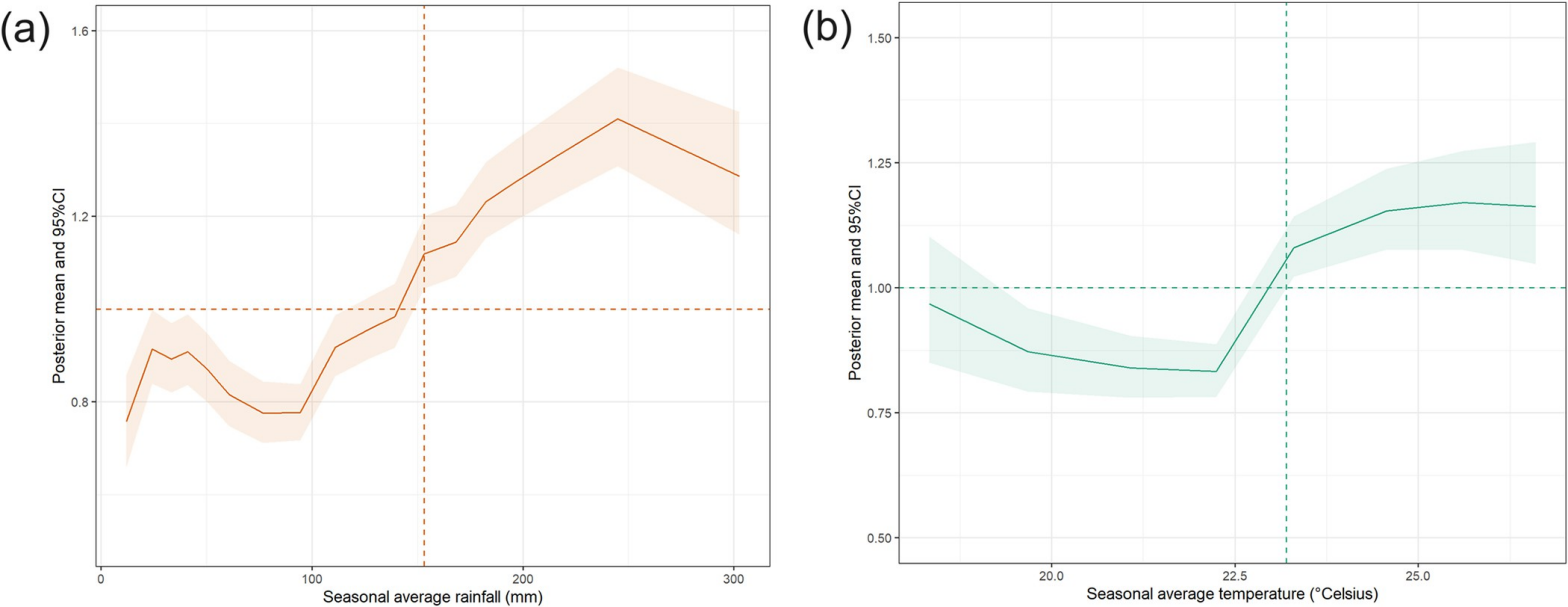
Posterior estimates for the nonlinear effect of (a) rainfall and (b) temperature: the solid line shows the posterior mean, and the ribbon describes the 95% Credible Intervals (CI).

We also found a clear nonlinear effect of seasonal average rainfall and temperature in the State of São Paulo during the period of study. The plots in Fig 4 highlight the posterior estimates for (a) rainfall, and (b) temperature, detecting an increase of the expected value of the vector index for values above 153.12 mm for rainfall and above 23.30°C for temperature.

**Fig 5:**
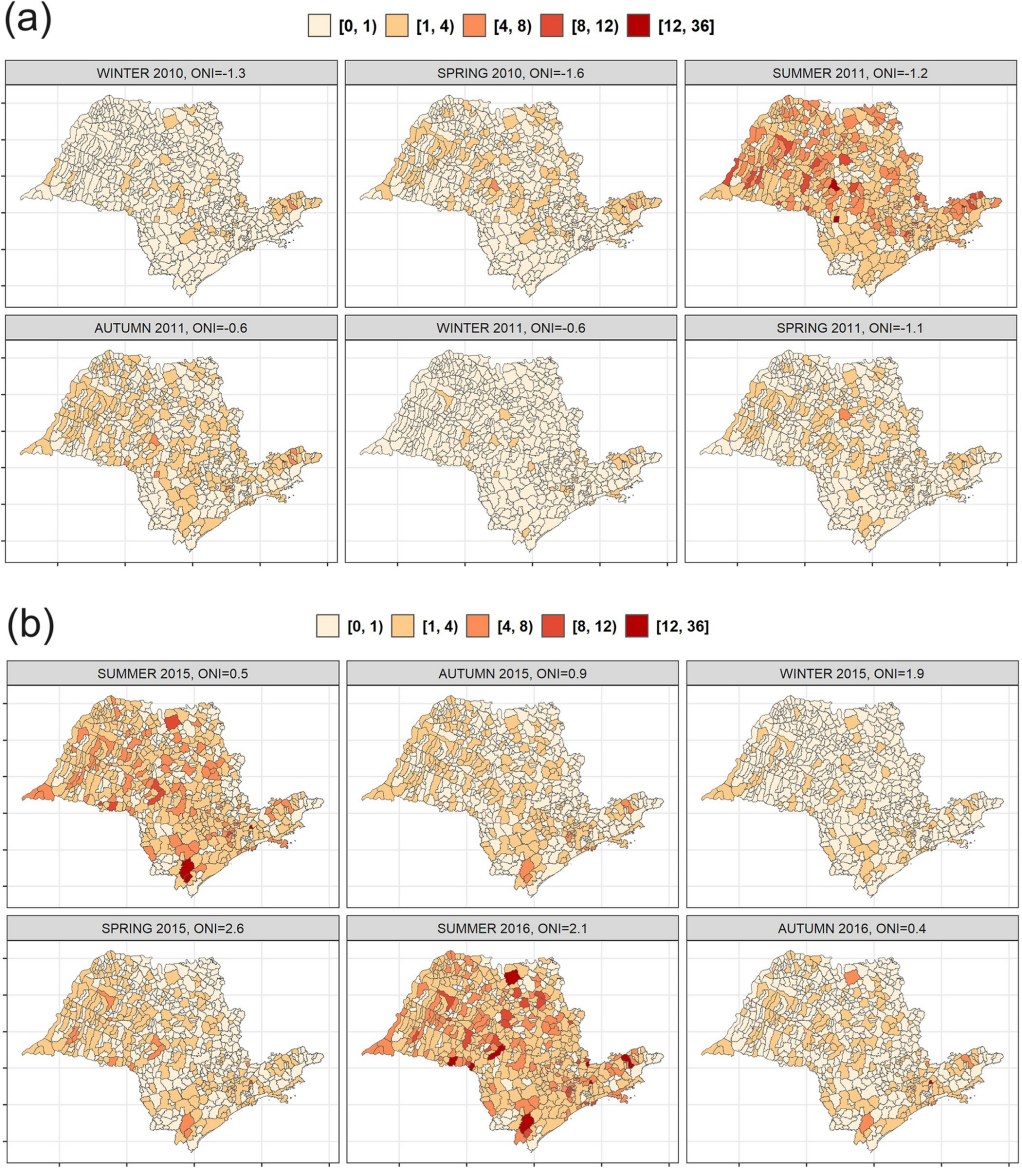
Posterior mean of the *Ae*. *aegypti* larval index for (a) La Niña event of 2010–2012 and (b) El Niño event of 2015–2016. *Source of map base layers: IBGE: https://www.ibge.gov.br/geociencias/cartas-e-mapas/mapas-estaduais.html). Open-source CC BY 4.0 license.

**Fig 6:**
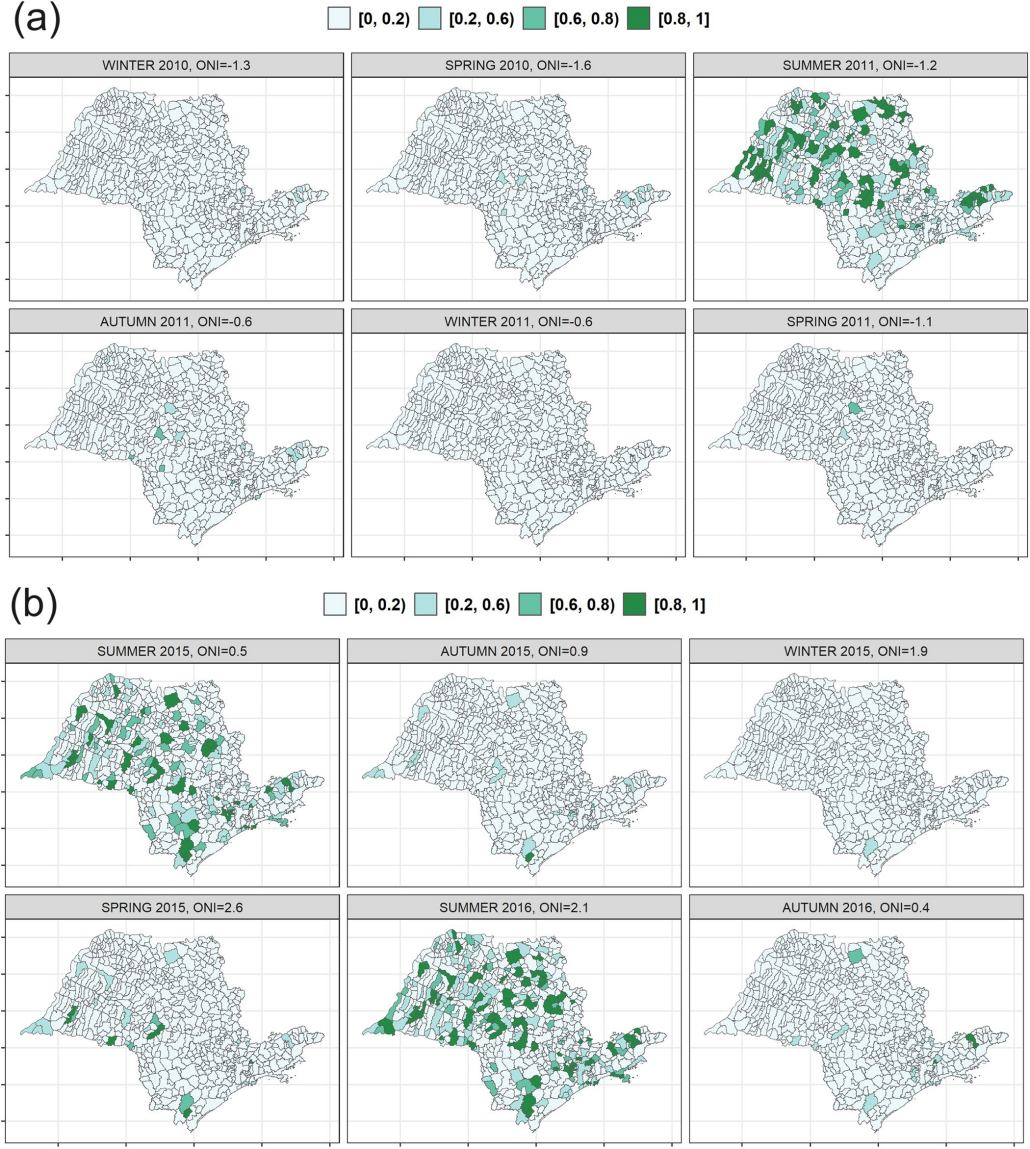
Exceedance probabilities that the *Ae*. *aegypti* larval index is ≥ 4 for La Niña event of 2010–2012 and El Niño event of 2015–2016. *Source of map base layers: IBGE: https://www.ibge.gov.br/geociencias/cartas-e-mapas/mapas-estaduais.html). Open-source CC BY 4.0 license.

In Fig 5 we present the estimated posterior means for the larval index for two ENSO events: (a) La Niña in 2010–2012, and (b) El Niño in 2015–2016. During La Niña event, we can notice a pronounced increase in the expected values of the larval index in the centre and north-west municipalities of the State, with some high spots in the south-east municipalities. However, during El Niño event, a diffuse increase in the expected values of the larval index is appreciable across the entire State, with marked hot spots in the south. This pattern is confirmed by Fig 6, which maps the probability for the expected vector index during La Niña event of 2010–2012 and El Niño event of 2015–2016 to be ≥ 4. We have chosen this threshold to identify a condition at risk for infestation in accordance with the indications of the Brazilian Ministry of Health [57], while values of the vector index less than 1 are considered satisfactory, and from 1 to 3.9 are identified as alert. These maps allow to identify the most vulnerable municipalities easily.

In S5 Fig we present also the estimated posterior means for the larval index for all the summer months during the study period, while S6 Fig displays the exceedance probabilities. Expected values on the larval index are noticeable higher in the centre and north-west part of the State of São Paulo, although for the summers hit by El Niño events (e.g., 2010 and 2016) the increase is appreciable across the entire State, confirming the findings presented in Figs 5 and 6.

## Discussion

Historical assessment of *Ae*. *aegypti* infestation levels in the State of São Paulo clearly shows an almost cyclical pattern over the years, with alternating high-risk areas based on information from LIRAa. This entomological condition, resulting from the action of different variables, may become a factor in the future management of major dengue epidemics in the State. Here, we focused on characterizing the influence of the ENSO regional event on the spatio-temporal *Ae*. *aegypti* infestation dynamics. We found a strong and significant coherence between the temporal pattern of mosquito abundance and El Niño phases and precipitation, showing the role that climate plays in driving infestation periodicity. Specifically, we identified a relationship between *Ae*. *Aegypti* infestation and the strength of the ENSO phenomenon: periods characterized by El Niño phase (from moderato to strong) were associated with a higher abundance of *Ae*. *aegypti* mosquitoes. At a regional scale, ENSO has been the most commonly studied driver of cyclic climate phenomena in human diseases [60]. ENSO events have been linked to recurrent outbreaks of mosquito-borne diseases such as dengue, malaria, and Rift Valley fever [15,60,61]. The occurrence of malaria epidemics in different nations and Rift Valley fever in East Africa appears to be influenced by higher-than-average rainfall associated with robust ENSO events, creating numerous expansive aquatic habitats conducive to the immature development of mosquito vectors [15].

In tropical South America, ENSO manifests as a recurrent climatic oscillation, occurring approximately every 3–7 years, as indicated by our analyses. It exerts a significant impact on the inter-annual variability of local climate across diverse geographical regions [1]. In Brazil, temperatures typically rise by an average of 0.5°C during El Niño, accompanied by severe droughts and negative anomalies in soil moisture, river discharges, and rainfall during this warm phase of the ENSO event. Consequently, El Niño years are characterized by warmer and drier conditions compared to no-Niño years [62].

The year 2015 was strongly influenced by El Niño, resulting in a strong heatwave that hit south-east Brazil in the months of September and October. Several cities recorded temperatures above 35°C, which may have favoured the proliferation of *Ae*. *aegypti* and its consequent increase in 2016. However, small increases in temperature resulting from climate change, associated with the high adaptive capacity of this mosquito, can also accelerate its infestation rates, and enable its spread to other regions previously unaffected [63].

The development time from egg to adulthood also depends heavily on climatic variables, especially the temperature: the hotter it is, the faster the cycle, and the greater the density of the adult population [42]. Studies show that mosquitoes can reduce their development time by up to 1.5 days when the temperature rises by 2°C [64]. Thus, global warming could increase the population of adult mosquitoes, and consequently increase larval infestation rates in the coming years. Optimal temperatures for development, longevity, and fecundity of *Ae*. *aegypti* mosquitoes are between 22°C and 32°C [65]. In our estimates we found that temperature had a positive effect on Breteau index from 23°C; given higher temperatures during extreme El Niño events, these conditions strongly favour the proliferation of *Ae*. *aegypti* in South America. The impact of ENSO on dengue epidemics is likely predominantly mediated by its warming influence on local temperatures. This, in turn, amplifies the replication of the dengue virus and influences the biting behaviour of the mosquito vector [27]. Our estimates highlighted that average seasonal rainfalls of more than 153.12 mm seem to have a notable impact on the larval index, leading to the accumulation of ample water in outdoor discarded receptacles, supporting the aquatic phase of mosquito development, which lasts 7–10 days [66]. However, mosquitoes can still breed in sites where there is water storage during droughts, due to its anthropophilic character and their ease of occupying containers inside homes. In northeast Thailand, for example, the 1987 dengue haemorrhagic fever epidemic occurred during a dry, hot season but stopped before the arrival of the rainy season [67].

Although the State of São Paulo as a whole has characteristics favourable to the proliferation of the vector (disordered population growth, highly urbanized population, predominantly hot climate), there are specific areas that have a greater tendency for infestation. We found a pronounced increase in the expected values on the vector index in the central and northern regions of the State, with marked hot spots of *Ae*. *Aegypti* proliferation in the south, especially during El Niño events. Additionally, we found that the larval index is positively associated with municipality wealth, and areas with larger inequalities are more vulnerable to vector abundance. This indicates that distinct socio-environmental traits, and even anthropogenic factors unique to each municipality, may impact levels of mosquito infestation. Several studies have shown a correlation between socioeconomic factors and elevated mosquito infestation levels [47]. In São Paulo, for example, this correlation is particularly pronounced in peripheral areas characterized by limited urban and health infrastructure and heightened social vulnerability [68]. While socioeconomic variables may not be the primary factors driving mosquito infestation, they do play a significant role in explaining a portion of the phenomenon. Therefore, it is crucial to devise public policies focused on interventions that target the improvement of these indicators. Such initiatives would yield substantial effects, leading to a reduction in mosquito abundance and, consequently, the transmission of arboviruses such as dengue. Mosquitoes display distinct habitat preferences depending on their species. While *Ae*. *aegypti* ‘s sensitivity to temperature and rainfall is widely acknowledged, in this study we found that ENSO climate pattern and also anthropogenic factors significantly influence their life cycle and geographic distribution. These instances illustrate how socioeconomic status within an area, along with climatic fluctuations, influences the interactions between mosquitoes and humans [69].

There were some limitations in this study. First, in regard to the data used for analysis, obtained exclusively from LIRAa. Larval surveys are carried out by different professionals in different municipalities, which allows varying degrees of effort spent on each collection, dependent on the diligence of each server in the performance of their duties. As a rule, the larval research procedure, which produces the results of LIRAa, must be carried out with the greatest possible adherence to the criteria, inspecting all potential breeding sites as well as all risk factors that may make for viable breeding and infestation conditions. But deficiency of equipment, material, and human resources are all factors in this type of survey, which can eventually affect the results of the indexes. It should be noted, however, that the LIRAa values used in our work all share the same criteria and serve as the official source of information for the various government agencies that study the entomological and epidemiological areas in which *Ae*. *aegypti* is involved. Second, the success of policy execution, including initiatives for dengue vector control, community involvement, and public health education, can significantly affect mosquito infestation. The effectiveness of these management strategies may be shaped by local practices and cultural factors and can exhibit variations over time. To mitigate the influence of interventions on model outcomes, improved historical survey data is essential. Nevertheless, our Bayesian approach offers valuable insights into the connections between ENSO patterns and mosquito infestation levels in various seasons, despite these constraints.

Our findings carry several implications for vector control policies. ENSO and local weather conditions act as reliable indicators of mosquito vector activity, along with seasonality and social inequalities, offering valuable insights for the development of early warning systems and spatial risk assessment, aiding in the prioritization of areas for more targeted vector control measures. Our findings elucidating the connection between El Niño events and *Ae*. *aegypti* infestations are particularly significant in light of recent explosive rise of dengue cases in Brazil, which has been associated, among the other, to El Niño events intensifying the heat and rainfall [69]. Thus, the emergence of moderate or strong El Niño conditions in Brazil could potentially serve as an advance indicator for alerting on the risk of arboviral epidemics in regions where the abundance of *Ae*. *aegypti* is high. Expanding this analysis to encompass all states and climate zones in Brazil would provide opportunities to capture the ENSO-driven variations in weather and *Aedes* vectors across the nation, offering ample lead time for planning source reduction initiatives and the allocation of resources to specific areas.

## Conclusions

We found a strong and significant association between the temporal pattern of mosquito abundance and El Niño phases and local weather, showing the fundamental role that climate plays in driving infestation periodicity. Specifically, we identified a relationship between *Ae*. *aegypti* infestation and the strength of the ENSO phenomenon active in that period. Our results showed that seasonal rainfall exceeding 153.12 mm appears to have a notable impact on vector indices, potentially leading to the accumulation of ample water in outdoor discarded receptacles, supporting the aquatic phase of mosquito development. We also found that seasonal temperature above 23.30°C favours the abundance of the *Ae*. *aegypti* larval index, along with increasing wealth inequalities. There are specific areas in the State of São Paulo that have a greater tendency for mosquito infestation, mainly those located in the central and northern regions of the state, with marked hot spots of abundance in the south, in particular during El Niño events. As the frequency of El Niño events is anticipated to rise due to climate change, our findings suggest a potential increase in arbovirus outbreaks. Furthermore, our findings emphasize the importance of considering global climatic oscillations in public health planning and El Niño events could serve as a valuable predictor, informing future modelling efforts and epidemiological investigations.

## Supporting information

S1 Fig Map of the State of São Paulo located in the Southeastern part of Brazil, displaying population density and wealth inequalities measured by the Gini index by municipality. *Source of map base layers: IBGE: https://www.ibge.gov.br/geociencias/cartas-e-mapas/mapas-estaduais.html). Open-source CC BY 4.0 license.
(TIF)

S2 Fig Proportion of municipalities of the State of São Paulo that did not carry out larval surveys over the years 2008–2018. (TIF)

S3 Fig Spatial distribution of the Breteau index during the spring months by year, with specification of the Oceanic Niño Index (ONI). State of São Paulo, 2008–2018. *Source of map base layers: IBGE: https://www.ibge.gov.br/geociencias/cartas-e-mapas/mapas-estaduais.html). Open-source CC BY 4.0 license.
(TIF)

S4 Fig Spatial variability of the Breteau index during the spring and summer months over the years 2008–2018. *Source of map base layers: IBGE: https://www.ibge.gov.br/geociencias/cartas-e-mapas/mapas-estaduais.html). Open-source CC BY 4.0 license.
(TIF)

S5 Fig Posterior mean of the *Ae*. *aegypti* larval index for the summer months by year with specification of the ONI value. *Source of map base layers: IBGE: https://www.ibge.gov.br/geociencias/cartas-e-mapas/mapas-estaduais.html). Open-source CC BY 4.0 license.
(TIF)

S6 Fig Exceedance probabilities that the *Ae*. *aegypti* larval index is ≥ 4 for the summer months over the years 2008–2018. *Source of map base layers: IBGE: https://www.ibge.gov.br/geociencias/cartas-e-mapas/mapas-estaduais.html). Open-source CC BY 4.0 license.
(TIF)

S1 Table Descriptive statistics of the Breteau index and local weather variables (temperature and rainfall) in the State of São Paulo over the years 2008–2018. (TIF)

S1 Text Additional details on the methods (model details, computation of the exceedance probabilities and model assessment). (PDF)
