# Supplementary figures and images for: Effects of the El Niño-Southern Oscillation and seasonal weather conditions on Aedes aegypti infestation in the State of São Paulo (Brazil): A Bayesian spatio-temporal study
Source: PLoS Negl Trop Dis. 2024 Sep 12;18(9):e0012397. doi: 10.1371/journal.pntd.0012397 (PMC11392405; doi:10.1371/journal.pntd.0012397)

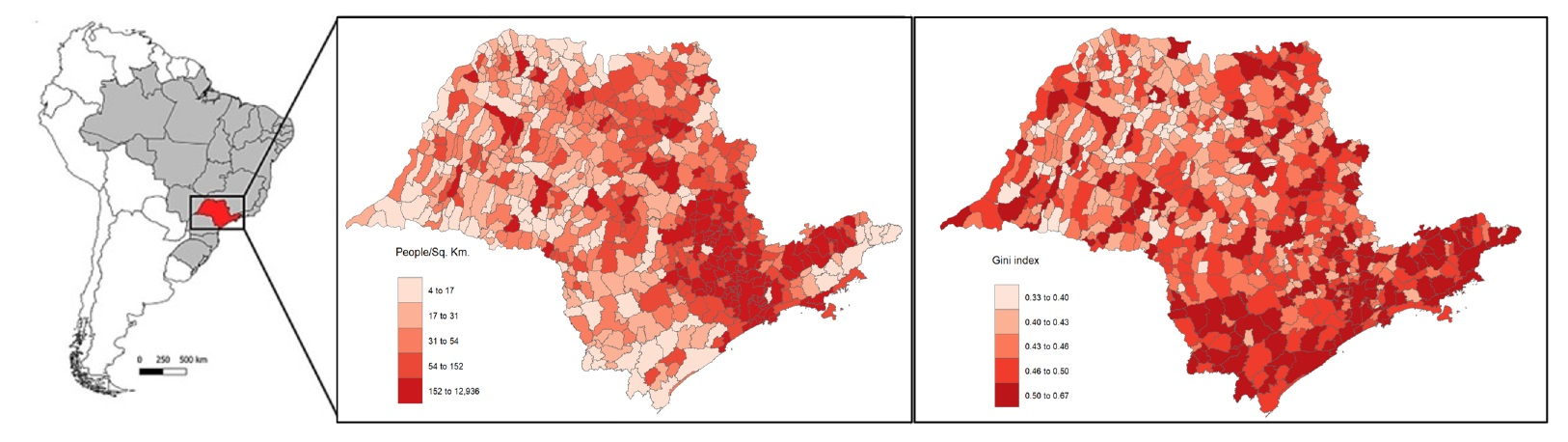

Supplement: S1 Fig — *Source of map base layers: IBGE: https://www.ibge.gov.br/geociencias/cartas-e-mapas/mapas-estaduais.html). Open-source CC BY 4.0 license. (TIF) [file pntd.0012397.s001.tif]

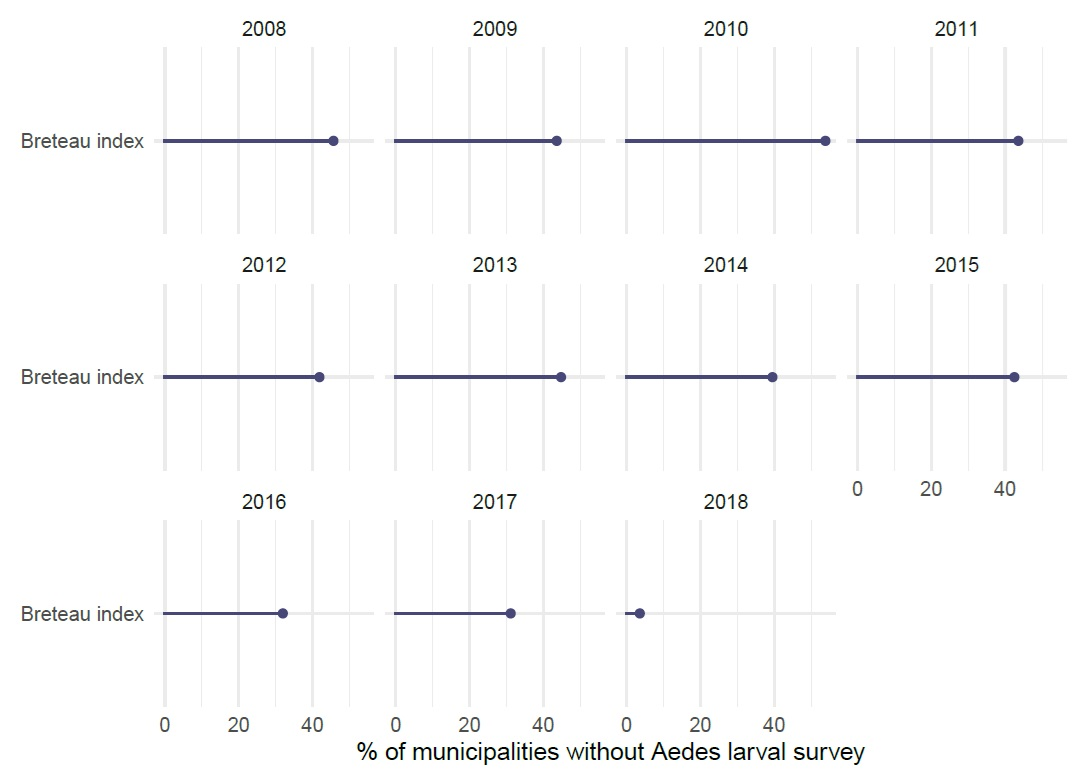

Supplement: S2 Fig — (TIF) [file pntd.0012397.s002.tif]

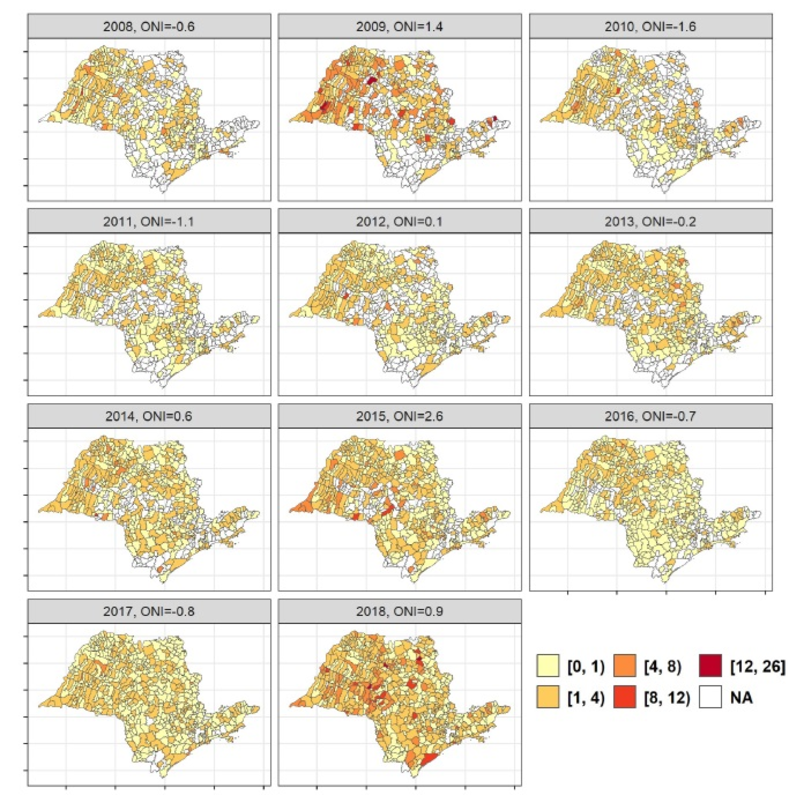

Supplement: S3 Fig — State of São Paulo, 2008–2018. *Source of map base layers: IBGE: https://www.ibge.gov.br/geociencias/cartas-e-mapas/mapas-estaduais.html). Open-source CC BY 4.0 license. (TIF) [file pntd.0012397.s003.tif]

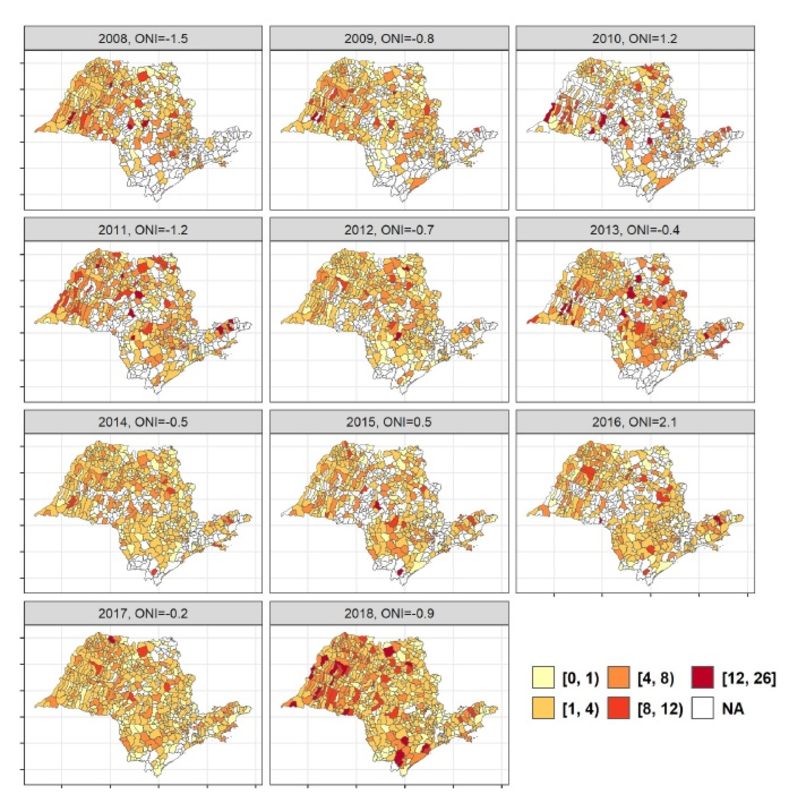

Supplement: S4 Fig — *Source of map base layers: IBGE: https://www.ibge.gov.br/geociencias/cartas-e-mapas/mapas-estaduais.html). Open-source CC BY 4.0 license. (TIF) [file pntd.0012397.s004.tif]

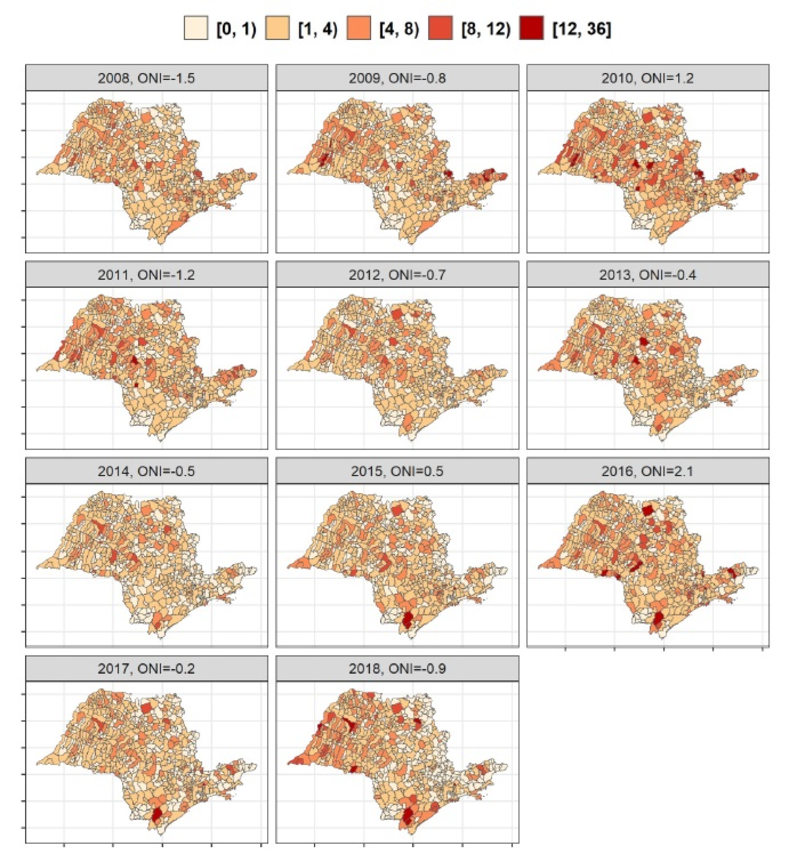

Supplement: S5 Fig — *Source of map base layers: IBGE: https://www.ibge.gov.br/geociencias/cartas-e-mapas/mapas-estaduais.html). Open-source CC BY 4.0 license. (TIF) [file pntd.0012397.s005.tif]

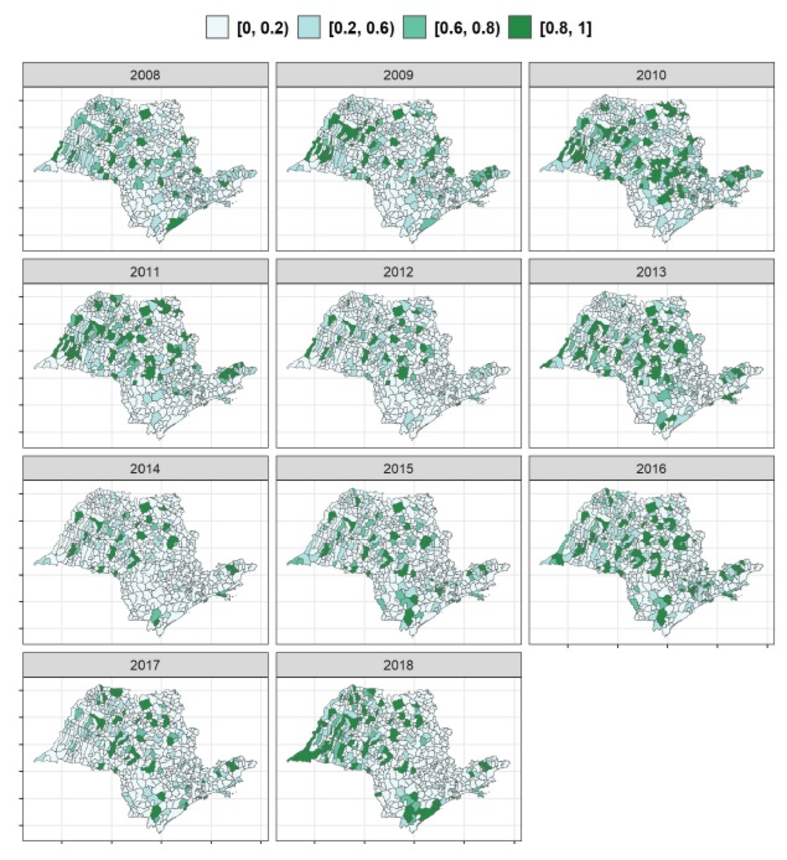

Supplement: S6 Fig — *Source of map base layers: IBGE: https://www.ibge.gov.br/geociencias/cartas-e-mapas/mapas-estaduais.html). Open-source CC BY 4.0 license. (TIF) [file pntd.0012397.s006.tif]

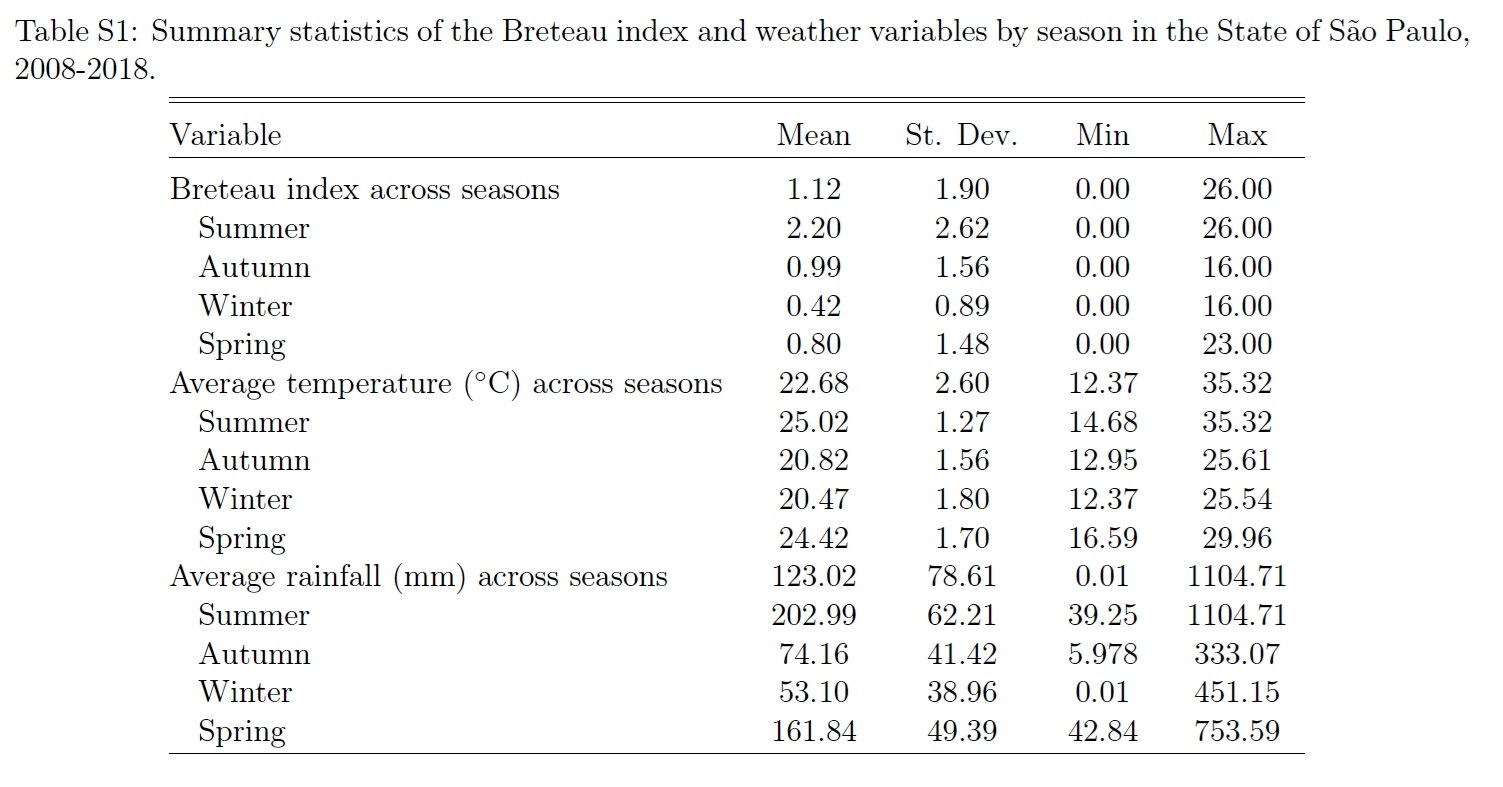

Supplement: S1 Table — (TIF) [file pntd.0012397.s007.tif]
