# Supplementary material for: Effects of the El Niño-Southern Oscillation and seasonal weather conditions on Aedes aegypti infestation in the State of São Paulo (Brazil): A Bayesian spatio-temporal study
Source: PLoS Negl Trop Dis. 2024 Sep 12;18(9):e0012397. doi: 10.1371/journal.pntd.0012397 (PMC11392405; doi:10.1371/journal.pntd.0012397)
Supplement: S1 Text — (PDF) [file pntd.0012397.s008.pdf]

Additional details on the methods

Model details

We specified a Bayesian spatio-temporal mixed model where the response is given by the larval index (i.e., Breteau index) collected over quarterly seasons in the 645 municipalities of the state of São Paulo from 2008 to 2018. Let y_{st} be the larval index collected across space ($s = 1, \ldots, S = 645$ municipalities) and time ($t = 1, \ldots, T = 44$ calendar quarter periods). We specified a Negative Binomial data model:

$$y_{st} \sim \text{NegBinom}(\mu_{st}, \varphi)$$

where φ is the size parameter. Note that we also tested a Poisson distribution model for the vector index with additional random effect modelled using an independent and identically distributed Gaussian variable to account for overdispersion, similarly to the model used in [1], but model assessment analyses played in favor of the Negative Binomial formulation. The process model was specified as follows:

$$\log(\mu_{st}) = \beta_0 + \sum_{k=2}^{3} \beta_k ONI_{tk} + f_1(Rain_{st}) + f_2(Temp_{st}) + \sum_{c=2}^{4} \phi_c Season_{tc} + \gamma_1 Density_{st} + \gamma_2 Gini_s + \delta_{st}, \quad (1)$$

were β_0 is the intercept, β_k is the coefficient associated with ENSO tracked by ONI (reference category: neutral phase), $f_1(\cdot)$ and $f_2(\cdot)$ capture nonlinearity in the effect of rainfall [$Rain$] and temperature [$Temp$] respectively, ϕ_c is the coefficient associated with calendar season [$Season$] (reference category: summer), γ_1 and γ_2 are the regression coefficients for population density [$Density$] and the Gini index [$Gini$] respectively, and δ_{st} is a residual random spatio-temporal effect.

To specify the nonlinear effect of the weather variables (i.e., rainfall and temperature) on the larval index, we binned their value into m groups, using equidistant quantiles in the probability space and we specified a first order random walk prior over the m groups. For example, considering temperature to demonstrate the model, we specified:

$$temp_{s(i+1)} - temp_{s(i)} \sim \mathcal{N}(0, \tau_{temp}^{-1}), \;\; i = 1, \ldots, m \quad (2)$$

where τ_{temp} is the precision (i.e., the inverse of the variance). We imposed a sum to zero constraints to make it identifiable, and for easier interpretation we scaled the model to have an average variance equal to 1.

We modelled the residual latent spatio-temporal term δ_{st} using a Gaussian Markov Random Field with zero mean; the covariance matrix assumes that within each time point the municipalities are linked through an intrinsic conditional autoregressive prior [2], which is a commonly used prior for the analysis of areal data incorporating dependence among locations through a spatial neighborhood structure [3]), while over time (i.e. between time points) the process evolves dynamically according to a first order autoregressive process with coefficient $\alpha < 1$ [4; 5].

For the (fixed) regression parameters we assume weakly informative Gaussian prior with mean 0 and variance 10, whereas penalising complexity (PC) priors [6] were used for the hyperparameters of the random walk structures, which penalise deviations from a simpler base model. Here, the distance from the base model is computed using the Kullback-Leibler distance, and penalization from the base model is performed at a constant rate on the distance [6; 7]. These priors are specified using probability statements. In particular, we specified that the probability for the standard deviations of the first order random walk models of being

greater than 1 is small, and is equal to 0.01. Default priors as specified in R-INLA were assumed for the other model parameters.

Computation of the exceedance probabilities

To identify unusual high values of the larval index we computed exceedance probabilities, defined as the probabilities of the expected values of the vector index, estimated for each municipality s at quarterly seasonal period t to be greater than a given threshold value c. Specifically, we set $c = 3.9$. In fact, based on the National Dengue Control Programme of the Brazilian Ministry of Health, a threshold of ≥ 4 identifies a condition at risk for *Ae. aegypti* infestation [8; 9]. To obtain the exceedance probabilities we used the built-in R-INLA function `inla.pmarginal()`. In particular, we first extracted the posterior marginal for each municipality at time point t on the orginal scale, then we calculated its probability to be larger than 3.9: $\Pr(\mu_{st} > 3.9) = 1 - \Pr(\mu_{st} \leq 3.9)$ [5].

Model assessment

In this section, we present the evaluation of the Bayesian models compared through the popular Deviance Information Criterion (DIC) [10], which is model fit statistics and represents a trade-off between model fit and complexity, and (ii) the cross-validated mean logarithmic score ($\overline{LS}$) [11], which assigns a numerical score to each model based on the posterior predictive distribution. Lower values indicate a better model fit.

Here, we describe the characteristics of the compared models by adopting the notation specified in the previous section, and letting $t = 1, \ldots, T = 44$ points on time and $s = 1, \ldots, S = 465$ municipalities. In detail, we specified:

M1: $\log(\mu_{st}) = \beta_0 + \lambda_1 Rain_{st} + \lambda_2 Temp_{st}$

M1a: $\log(\mu_{st}) = \beta_0 + \lambda Rain_{st} + f(Temp_{st})$

M1b: $\log(\mu_{st}) = \beta_0 + f(Rain_{st}) + \lambda Temp_{st}$

M1c: $\log(\mu_{st}) = \beta_0 + f_1(Rain_{st}) + f_2(Temp_{st})$

M2: $\log(\mu_{st}) = \beta_0 + \sum_{k=2}^{3} \beta_k ONI_{tk}$

M3: $\log(\mu_{st}) = \beta_0 + \sum_{k=2}^{3} \beta_k ONI_{tk} + f_1(Rain_{st}) + f_2(Temp_{st}) + \sum_{c=2}^{4} \phi_c Season_{tc}$

M4: $\log(\mu_{st}) = \beta_0 + \sum_{k=2}^{3} \beta_k ONI_{tk} + f_1(Rain_{st}) + f_2(Temp_{st}) + \sum_{c=2}^{4} \phi_c Season_{tc} + \gamma_1 Density_{st} + \gamma_2 Gini_s$

M5: $\log(\mu_{st}) = \beta_0 + \sum_{k=2}^{3} \beta_k ONI_{tk} + f_1(Rain_{st}) + f_2(Temp_{st}) + \sum_{c=2}^{4} \phi_c Season_{tc} + \gamma_1 Density_{st} + \gamma_2 Gini_s + \delta_{st}$

In Table S2 we present the DIC and the mean logarithmic scores, and in Table S3 the results for the (fixed) regression coefficients under the different models.

Table S2: Model fit statistic (DIC) and cross-validate mean logarithmic score ($\overline{LS}$)

Model	DIC	$\overline{LS}$
M1	49299.00	1.409
M1a	49213.83	1.407
M1b	49176.11	1.406
M1c	49094.27	1.403
M2	50663.86	1.448
M3	47727.54	1.364
M4	47307.83	1.352
M5	39105.13	1.197

Table S3: Posterior mean (95% Credible Intervals [95%CI]) for the fixed regression parameters associated with the *Ae. aegypti* larval index by model. Estimates are on the natural scale. Note that continuous variables included with linear effect into the models were standardised, thus their interpretation is on the scale of the standard deviation.

Variables	M1	M2	M3	M4	M5
**ONI**					
Neutral	-	1	1	1	1
La Niña	-	1.32 (1.23 to 1.42)	1.02 (0.95 to 1.10)	1.07 (1.00 to 1.15)	1.06 (1.00 to 1.13)
El Niño	-	1.47 (1.37 to 1.58)	1.30 (1.21 to 1.39)	1.30 (1.21 to 1.40)	1.30 (1.23 to 1.37)
**Rainfall**					
Linear	1.43 (1.39 to 1.47)	-			
Nonlinear			$f_1(Rain_{st})$	$f_1(Rain_{st})$	$f_1(Rain_{st})$
**Temperature**					
Linear	1.17 (1.13 to 0.20)	-			
Nonlinear			$f_2(Temp_{st})$	$f_2(Temp_{st})$	$f_2(Temp_{st})$
**Season**					
Summer	-	-	1	1	1
Autumn	-	-	0.78 (0.69 to 0.89)	0.93 (0.82 to 1.06)	0.81 (0.70 to 0.93)
Winter	-	-	0.31 (0.27 to 0.36)	0.37 (0.33 to 0.43)	0.32 (0.28 to 0.37)
Spring	-	-	0.40 (0.38 to 0.42)	0.41 (0.38 to 0.43)	0.37 (0.35 to 0.39)
**Pop. density**	-	-	-	1.02 (1.00 to 1.04)	1.00 (0.88 to 1.13)
**Gini index**	-	-	-	1.27 (1.24 to 1.30)	1.42 (1.31 to 1.54)

References

1. Liyanage P, Tozan Y, Overgaard HJ, Tissera HA, Rocklöv J. Effect of El Niño–Southern Oscillation and local weather on Aedes dvector activity from 2010 to 2018 in Kalutara district, Sri Lanka: a two-stage hierarchical analysis. The Lancet Planetary Health. 2022;6(7):e577–e585.

2. Besag J. Spatial interactions and the statistical analysis of lattice systems (with discussion). Journal of the Royal Statistical Society: Series B. 1974;36:192–236.

3. MacNab YC. Revisiting Gaussian Markov random fields and Bayesian disease mapping. Statistical Methods in Medical Research. 2023;32(1):207–225.

4. Martins TG, Simpson D, Lindgren F, Rue H. Bayesian computing with INLA: new features. Computational Statistics & Data Analysis. 2013;67:68–83.

5. Blangiardo M, Cameletti M. Spatial and spatio-temporal Bayesian models with R-INLA. John Wiley & Sons; 2015.

6. Simpson D, Rue H, Riebler A, Martins TG, Sørbye SH. Penalising model component complexity: A principled, practical approach to constructing priors. Statistical science. 2017;32(1):1–28.

7. Gómez-Rubio V. Bayesian inference with INLA. CRC Press; 2020.

8. Ministério da Saúde, Brasil. Levantamento rápido de índices para Aedes aegypti–LIRAa para vigilância entomológica do Aedes aegypti no Brasil: metodologia para avaliação dos índices de Breteau e Predial e tipo de recipientes; 2013.

9. Costa SdSB, Branco MdRFC, Aquino Junior J, Rodrigues ZMR, Queiroz RCdS, Araujo AS, et al. Spatial analysis of probable cases of dengue fever, chikungunya fever and zika virus infections in Maranhao State, Brazil. Revista do Instituto de Medicina Tropical de São Paulo. 2018;60:e62.

10. Spiegelhalter DJ, Best NG, Carlin BP, Van Der Linde A. Bayesian measures of model complexity and fit. Journal of the Royal Statistical Society Series B: Statistical Methodology. 2002;64(4):583–639.

11. Schrödle B, Held L, Riebler A, Danuser J. Using integrated nested Laplace approximations for the evaluation of veterinary surveillance data from Switzerland: a case-study. Journal of the Royal Statistical Society Series C: Applied Statistics. 2011;60(2):261–279.
